# Critical care delivery across health care systems in low-income and low-middle-income country settings: A systematic review

**DOI:** 10.7189/jogh.13.04141

**Published:** 2023-12-01

**Authors:** Emily S Bartlett, Andrew Lim, Sean Kivlehan, Lia I Losonczy, Srinivas Murthy, Richard Lowsby, Alfred Papali, Madiha Raees, Bhavna Seth, Natalie Cobb, Jason Brotherton, Enrico Dippenaar, Gaurav Nepal, Gentle S Shrestha, Shih-Chiang E Kuo, J Ryan Skrabal, Margaret Davis, Cappi Lay, Sojung Yi, Michael Jaung, Brandon Chaffay, Nana Sefa, Marc LC Yang, P Andrew Stephens, Amir Rashed, Nicole Benzoni, Bernadett Velasco, Neill KJ Adhikari, Teri Reynolds

**Affiliations:** 1 Department of Emergency Medicine, University of New Mexico, Albuquerque, New Mexico, USA; 2 Section of Critical Care Medicine, Virginia Mason Franciscan Health, Seattle, Washington, USA; 3 Department of Emergency Medicine, Brigham and Women’s Hospital, Boston, Massachusetts, USA; 4 Harvard Humanitarian Initiative, Cambridge, Massachuesetts, USA; 5 Department of Emergency Medicine, Department of Anaesthesia and Critical Care Medicine, George Washington University Medical Center, Washington, District of Columbia, USA; 6 Department of Pediatrics, University of British Columbia, Vancouver, British Columbia, Canada; 7 Department of Critical Care Medicine, Department of Emergency Medicine, Mid Cheshire Hospitals National health Service Foundation Trust, Cheshire, UK; 8 Pulmonary and Critical Care Medicine, Atrium Health, Pineville, North Carolina, USA; 9 Division of Critical Care Medicine, Department of Anaesthesiology and Critical Care Medicine, The Children’s Hospital of Philadelphia, Philadelphia, Pennsylvania, USA; 10 Division of Pulmonary and Critical Care Medicine, Johns Hopkins Medicine, Baltimore, Maryland, USA; 11 Division of Pulmonary, Critical Care and Sleep Medicine, University of Washington, Seattle, Washington, USA; 12 Department of Internal Medicine and Paediatrics, Africa Inland Church Kijabe Hospital, Kijabe Kenya; 13 Department of Critical Care Medicine, University of Pittsburgh, Pittsburgh, Pennsylvania, USA; 14 University of Cape Town, Cape Town, South Africa; 15 Ministry of Health and Population, Kathmandu, Nepal; 16 Department of Critical Care Medicine, Tribhuvan University Teaching Hospital, Maharajgunj, Kathmandu, Nepal; 17 The Johns Hopkins Bloomberg School of Public Health, Baltimore, Maryland, USA; 18 Department of Emergency Medicine, George Washington University, Washington, District of Columbia, USA; 19 Department of Emergency Medicine, University of Washington, Seattle, Washington, USA; 20 Department of Neurosurgery, Department of Emergency Medicine, The Mount Sinai Hospital, New York, New York, USA; 21 Stanford University, Stanford, California, USA; 22 Department of Emergency Medicine, Baylor College of Medicine, Houston, Texas, USA; 23 Department of Emergency Medicine, Department of Critical Care, Medstar Washington Hospital Center, Washington, District of Columbia, USA; 24 Accident and Emergency Medicine, The Chinese University of Hong Kong Faculty of Medicine, Hong Kong; 25 Department of Emergency Medicine, Intensive Care & Resuscitation, Cleveland Clinic Foundation, Cleveland, Ohio, USA; 26 Albert Einstein College of Medicine, New York, New York, USA; 27 Critical Care Medicine, Virginia Mason Franciscan Health, Silverdale, Washington, USA; 28 Department of Emergency Medicine, East Avenue Medical Center, Quezon City, National Capital Region, Philippines; 29 Department of Critical Care Medicine, Sunnybrook Health Sciences Centre and University of Toronto, Toronto, Ontario, Canada; 30 Department of Integrated Health Services, World Health Organization, Geneva, Switzerland

## Abstract

**Background:**

Prior research has demonstrated that low- and low-middle-income countries (LLMICs) bear a higher burden of critical illness and have a higher rate of mortality from critical illness than high-income countries (HICs). There is a pressing need for improved critical care delivery in LLMICs to reduce this inequity. This systematic review aimed to characterise the range of critical care interventions and services delivered within LLMIC health care systems as reported in the literature.

**Methods:**

A search strategy using terms related to critical care in LLMICs was implemented in multiple databases. We included English language articles with human subjects describing at least one critical care intervention or service in an LLMIC setting published between 1 January 2008 and 1 January 2020.

**Results:**

A total of 1620 studies met the inclusion criteria. Among the included studies, 45% of studies reported on pediatric patients, 43% on adults, 23% on infants, 8.9% on geriatric patients and 4.2% on maternal patients. Most of the care described (94%) was delivered in-hospital, with the remainder (6.2%) taking place in out-of-hospital care settings. Overall, 49% of critical care described was delivered outside of a designated intensive care unit. Specialist physicians delivered critical care in 60% of the included studies. Additional critical care was delivered by general physicians (40%), as well as specialist physician trainees (22%), pharmacists (16%), advanced nursing or midlevel practitioners (8.9%), ambulance providers (3.3%) and respiratory therapists (3.1%).

**Conclusions:**

This review represents a comprehensive synthesis of critical care delivery in LLMIC settings. Approximately 50% of critical care interventions and services were delivered outside of a designated intensive care unit. Specialist physicians were the most common health care professionals involved in care delivery in the included studies, however generalist physicians were commonly reported to provide critical care interventions and services. This study additionally characterised the quality of the published evidence guiding critical care practice in LLMICs, demonstrating a paucity of interventional and cost-effectiveness studies. Future research is needed to understand better how to optimise critical care interventions, services, care delivery and costs in these settings.

**Registration:**

PROSPERO CRD42019146802.

Critical illness represents any immediately life-threatening disease or injury that, if left untreated, can lead to death [1]. It is not specific to a single disease category, patient population or age group and may be encountered throughout the health care system, including intensive care unit (ICU) settings, emergency care units, operating rooms, post-anesthetic care units, wards and pre-hospital settings [2]. Critical care may be defined as treating severely ill patients irrespective of care setting, care provider, or specific technologies utilised [3]. The unmet need for timely and high-quality critical care interventions and services is especially pressing in low- and low-middle-income country (LLMIC) settings [4]. The burden of critical illness is often higher in LLMICs than in high-income countries (HICs), and there is also a higher risk of death and loss of disability-adjusted life years (DALYs) from critical illness in lower-income settings [3,5-9]. The provision of critical care in LLMICs is challenging due to more limited resources than in HICs. However, many critical care interventions and services, such as intravenous fluids, antibiotics, haemorrhage control, close monitoring of patients and supplemental oxygen, are feasible to deliver in resource-constrained settings[10].

Effective initial care for critically ill patients requires recognition and resuscitation, followed in some cases by access to definitive care, which necessitates temporal and spatial alignment of critical care interventions and services with patient needs and location[2,11]. Given the significant heterogeneity in the distribution of resources within and between health care systems worldwide, models for critical care intervention and service delivery tailored to local circumstances and resources are required [2,12,13]. For example, due to the lack of ICU capacity in LLMICs, critically ill patients may be cared for in non-traditional environments, such as emergency departments, hospital wards, emergency care units or pre-hospital settings. Therefore, understanding how, where and by whom critical care is currently delivered in LLMICs is essential to strengthening the delivery of critical care across the breadth of the health care system.

The primary aim of this systematic review was to characterise the range of critical care interventions and services delivered across health care systems in LLMICs, as reported in the literature. We report the health service location in which critical care is delivered, the health care professionals involved, the populations treated, and the disease or syndrome categories addressed by critical care interventions and services.

## METHODS

The methods for this review have been published previously [14]. In summary, controlled vocabulary terms and text words related to critical care in LLMICs were developed. The search strategy was implemented in PubMed/MEDLINE, EMBASE, Web of Science and PROSPERO databases (**Online Supplementary Document**). We restricted search results to citations in English pertaining to humans published from 1 January 2008 to 1 January 2020. The Stanford University Office for Human Subjects Research and Institutional Review Board reviewed and exempted this study on 20 May 2020. Reviewers were unblinded to authors, as well as institutional details of all included citations.

The eligibility criteria were set as: design (original peer-reviewed research or systematic review), setting (LLMICs as defined by the 2016 World Bank classification [15]), participants (any age group), and interventions (at least one critical care intervention or service described). The list of qualifying critical care interventions and services was selected to reflect a broad definition of critical care informed by the World Health Organization (WHO) Emergency Care Systems Framework [16]. Care delivered in operating rooms as part of a surgical procedure was excluded. Care delivered by lay providers was also excluded.

We uploaded citations generated from this search strategy to Covidence v 2.0 (Covidence, Melbourne, Australia) and completed abstract and full-text screening. We completed data extraction utilising Redcap v11.2.2 [17,18].

Reviewers were required to have medical training at a minimum level of a senior medical student and completed an orientation regarding the inclusion and exclusion criteria and the process for data extraction. Two reviewer agreement was required for the inclusion of each abstract and full-text article. Discrepancies were resolved by a third reviewer, who made the final determination regarding inclusion. A single reviewer completed data extraction for each study meeting inclusion criteria. We randomly selected five percent of included studies to be audited by lead investigators to ensure data extraction quality and consistency. Approximately 30 individuals contributed to abstract and full text review and data extraction stages. We calculated inter-rater reliability by percent agreement.

We completed data analysis using descriptive statistics utilising Microsoft Excel v16.75.2 (Microsoft Corporation, Redmond, Washington, USA). Data visualisation for Figure 2 was created utilising ArcGIS v 10.6.1(Esri, Redlands, California, USA) and data visualisation for Figure S1 in the **Online Supplementary Document**) was created utilising Tableau (Tableau Software, Seattle, Washington, USA). Where applicable, the review is reported according to PRISMA guidance [19].

## RESULTS

### Characteristics of included studies

**Figure 1:**
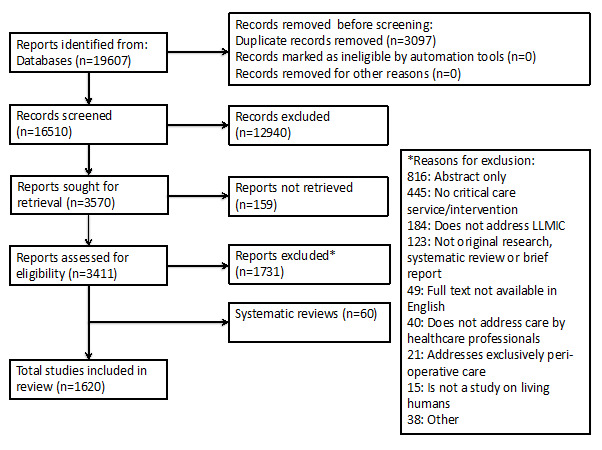
Study selection.

16 510 original records were screened for inclusion; 12 940 records were excluded after abstract screening. Of the 3570 full-text reports, 159 could not be accessed. Ultimately, 1620 studies were included in the review (**Figure 1**). Raw inter-rater reliability for data extraction was 0.99 for audited studies. The majority of included studies had an observational study design (81%), while 93 studies (5.7%) were randomised controlled trials (**Table 1**). Further, 10% (163 studies) reported on the costs or economics of critical care interventions and services described.

**Table 1:** Characteristics of included studies

Category	n (%)
**Study design (n = 1620)**	
Cohort	624 (38.0)
Cross-sectional	593 (37.0)
Randomised controlled trial	93 (5.7)
Case-control	88 (5.4)
Educational	62 (3.8)
Qualitative study	24 (1.5)
Mixed methods	16 (1.0)
Other	121 (7.5)
Population (n = 2288)*	
Paediatric	1027 (45.0)
Infant	520 (23.0)
Adult	976 (43.0)
Geriatric	203 (8.9)
Maternal	97 (4.2)
Other unspecified	188 (8.2)
**Healthcare workers (n = 1550)†**	
Specialist doctor	439 (60.0)
General doctor	322 (44.0)
Specialist doctor trainee	164 (22)
Medical student	9 (1.2)
Advanced nursing/midlevel practitioner	65 (8.9)
Nurse	368 (50.0)
Nursing student	10 (1.4)
Respiratory therapist	23 (3.1)
Pharmacist	28 (16.0)
Ambulance provider	24 (3.3)
Non-clinical providers	6 (0.8)
Other	92 (13.0)

*Overlapping categories permitted. Percentages reported with total included studies as denominator. The following definitions were utilised: pediatric (>1 y old and <18 y old), infant (<1 y old), adult (≥18 y old), geriatric (>65 y old), maternal (patient of any age during pregnancy and childbirth or within 42 d of termination of pregnancy).

†Overlapping categories permitted. Percentages reported with total included studies reporting at least one type of care provider as the denominator.

### Patient populations

Included studies most commonly reported on pediatric (n = 1027, 43%) and adult (n = 976, 43%) patients. Infants (defined as less than one year old) were the next most commonly included patient group (n = 520, 23%). A smaller percentage reported on geriatric (defined as more than 65 years old, n = 203, 8.9%) and maternal patient populations (n = 97, 4.3%) (**Table 1**).

### Healthcare workers

Among health care worker cadres described, specialist doctors (defined as physicians with critical care or other specialty training) provided care in 60% of included studies (n = 439), while general doctors provided care in 44% of included studies (n = 322). Nurses were explicitly described as providing care in 50% of included studies (n = 368). Specialist doctor trainees (n = 164, 22%), pharmacists (n = 28, 16%), advanced nursing or midlevel practitioners (n = 65, 8.9%), ambulance providers (n = 24, 3.3%) and respiratory therapists (n = 23, 3.1%) were also reported to provide care in a smaller proportion of the studies (**Table 1**). In 890 studies (55% of included studies), the type of care provider was not specified.

### Countries

**Figure 2:**
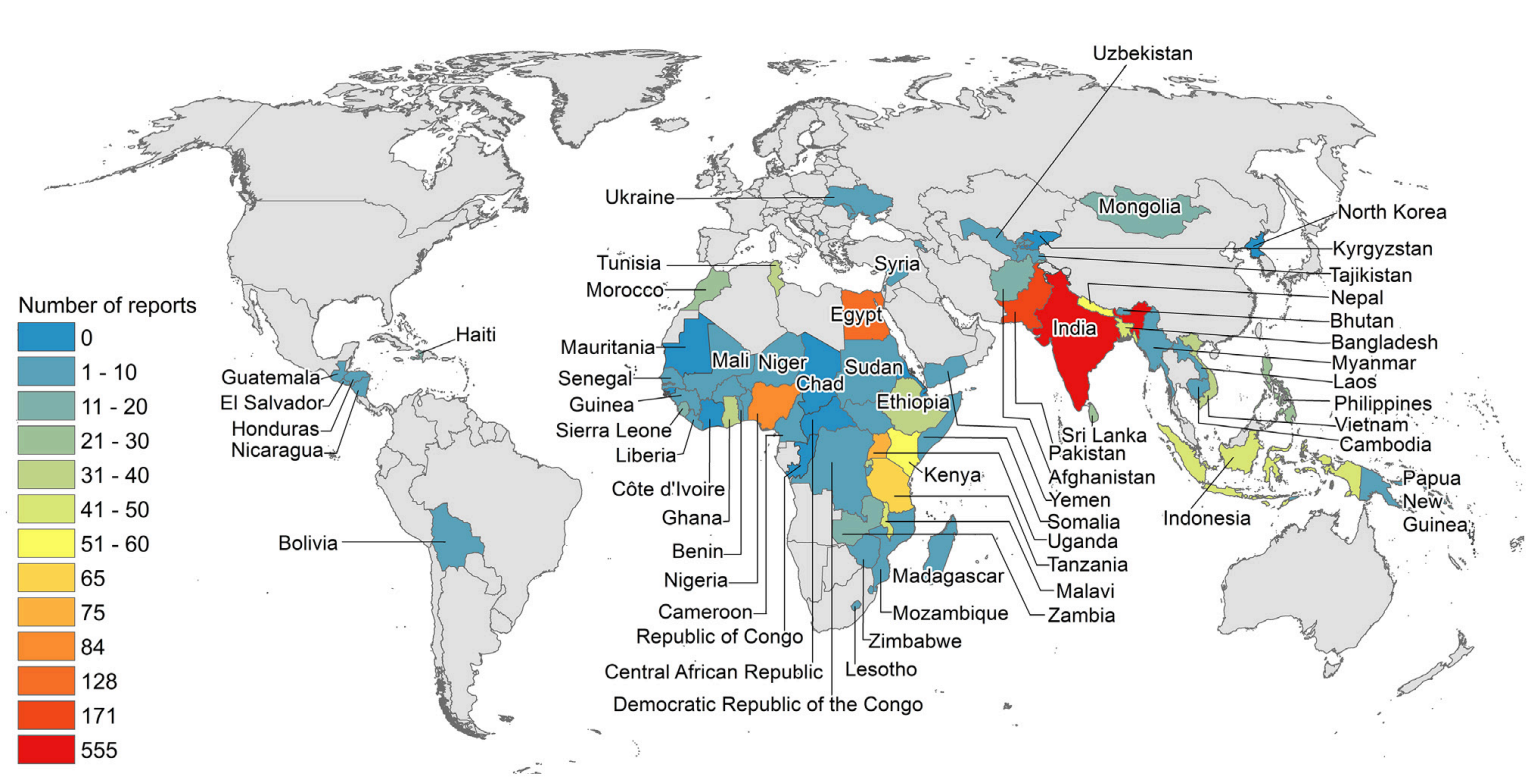
Countries reported on in included studies.

India was the LLMIC most frequently reported on (n = 555, 31%), followed by Pakistan (n = 171, 9.4%). There were no studies reporting on 17 LLMICs (20% of all LLMICs) (**Figure 2**).

### Care settings

Most of the critical care described was delivered in in-hospital settings (94%). Overall, approximately half (51%) of the care described occurred in an ICU, as self-defined, whereas the emergency department and non-ICU hospital ward each comprised approximately 13% of the care settings described (Figure S1 in the **Online Supplementary Document**). All combined out-of-hospital care settings, including clinic, field, ambulance and temporary health response unit settings, comprised 6.2% of reported care settings.

### Critical care interventions and services

Among 6266 total critical care interventions and services reported, respiratory interventions (20%) were most frequently described. Diagnostic modalities and interventions for haemodynamic instability or organ dysfunction each comprised 15% of reported interventions and services, followed by nursing or close monitoring (13%), and multi-system processes (11%) (e.g. prognosis-based advanced care planning, care pathways and critical illness severity stratification). Critical care education and capacity-building represented 5.1% of reported interventions/services (**Table 2**).

**Table 2:** Critical care interventions and services provided

Category	n (%)
**Respiratory interventions**	1240 (20.0)
Mechanical ventilation, invasive	439 (7.0)
Support of respiratory insufficiency/failure	351 (5.6)
Mechanical ventilation, non-invasive	133 (2.1)
Advanced invasive airway management, non-surgical	99 (1.6)
Oxygen delivery, simple (face mask, nasal prongs)	86 (1.4)
Non-invasive airway management	67 (1.1)
Advanced surgical airway management	53(0.8)
Oxygen delivery, high flow	12 (0.2)
**Diagnostic modalities**	927 (15.0)
Laboratory and other rapid results reporting, including point-of-care diagnostics	309 (4.9)
Microbiology and other infectious rapid results reporting	207 (3.3)
Utilisation of targeted diagnostic strategy to establish timely etiology in critical illness	152 (2.5)
Basic radiography	107 (1.7)
Critical care ultrasound	74 (1.2)
Computed tomography	64 (1.0)
Magnetic resonance imaging	14 (0.2)
**Interventions for haemodynamic instability/organ dysfunction**	919 (15.0)
Support of haemodynamic instability and management of acute life-threatening organ dysfunction	267 (4.3)
Administration of vasopressors and inotropes	165 (2.6)
Intravenous fluid resuscitation	150 (2.4)
Blood product transfusion	117 (1.9)
Cardiopulmonary resuscitation, basic	59 (0.9)
Advanced cardiac life support resuscitation	46 (0.7)
Massive haemorrhage control	26 (0.4)
Advanced trauma resuscitation/ trauma care checklist	24 (0.4)
Advanced blood replacement therapies (e.g. plasmapheresis, plasma exchange, exchange transfusion)	22 (0.4)
Anti-arrhythmic medication administration	18 (0.3)
Targeted temperature management and hyperthermia/hypothermia management	16 (0.3)
Spinal immobilisation	7 (0.1)
Extracorporeal membrane oxygenation	2 (0.03)
**Nursing and close monitoring**	787 (13.0)
Frequent monitoring, surveillance and recording of clinical parameters	358 (5.7)
Acuity-based triage/performance of focused assessment for critical illness state	263 (4.2)
Critical care nursing services	130 (2.1)
Titration of advanced parenteral therapeutics	22 (0.4)
Foetal monitoring	14 (0.2)
**Additional targeted therapies**	747 (11.9)
Antibiotic administration in critical illness	244 (3.9)
Monitoring and treatment of critical electrolyte/metabolic/acid-base derangements	133 (2.1)
Renal replacement therapy	107 (1.7)
Treatment of severe infections other than intravenous fluids and antibiotics	65 (1.0)
Emergent poisoning detoxification/antidote	60 (1.0)
Nutrition management in critically ill/injured patients	50 (0.8)
Provision of prophylaxis associated with critical illness	29 (0.5)
Acute reperfusion therapy, cardiac	28 (0.5)
Advanced burn care	27 (0.4)
Acute reperfusion therapy, Venous thromboembolism	4 (0.1)
**Multi-system processes**	661 (11.0)
Critical care triage/care pathways systems, clinical illness severity and/or risk stratification	403 (6.4)
Coordination of specialist services for multi-system illness	105 (1.7)
Prognosis-based advanced care planning	83 (1.3)
Health information systems	49 (7.8)
Critical care level crisis management	21 (0.3)
**Neurological interventions**	233 (3.7)
Acute medical stabilisation of critical neurologic illness	114 (1.8)
Analgesia and sedation	66 (1.1)
Acute surgical stabilisation of critical neurologic illness	37 (0.6)
Acute management of agitation/delirium	11 (0.2)
Acute reperfusion therapy, neurologic	5 (0.08)
**Obstetrical care**	141 (2.2)
Obstetric critical care	141 (2.2)
**Other invasive procedures**	133 (2.1)
Advanced vascular access (central venous catheters, arterial lines, pulmonary artery catheters)	81 (1.3)
Peripheral venous cannulation	34 (0.5)
Thoracic invasive procedures (thoracostomy, pleural drain placement, thoracentesis, pericardiocentesis, thoracotomy)	17 (0.3)
Intra-osseous access	1 (0.02)
**Other**	359 (5.7)
Critical care education and capacity building	318 (5.1)
Critical care pharmacy services	41 (0.7)
Other critical care intervention/service delivery	119 (1.9)

*Overlapping categories permitted.

### Disease and syndrome categories

Non-communicable diseases comprised 32% of disease categories addressed while infectious diseases comprised 27% (**Table 3**). Reproductive, maternal and newborn health represented the single most common disease category addressed (10%), followed by an integrated approach to respiratory distress and respiratory failure (8.8%). The most common individual infectious disease processes cited included sepsis (7.1%) and respiratory infections (4.5%). Malaria was addressed in 1.4% of studies, tuberculosis in 0.7% and human immunodeficiency virus (HIV) in 0.6%. The most common non-communicable disease processes addressed included injuries, envenomation and toxic exposures (7.0%), respiratory diseases (5.8%), cardiovascular diseases (5.0%) and neurologic disorders (4.4%).

**Table 3:** Disease and syndrome processes addressed by included studies

Disease process	n (%)*
**Infectious disease**	1033 (27)
Integrated approach to sepsis†	271 (7.1)
Infectious disease, not otherwise specified	228 (6.0)
Respiratory infections	172 (4.5)
Other infectious disease processes	137 (3.6)
Disease prevention and surveillance	55 (1.4)
Malaria	53 (1.4)
Neglected tropical diseases	37 (1.0)
Gastrointestinal infections	28 (0.7)
Tuberculosis	25 (0.7)
HIV	24 (0.6)
Viral hepatitides	3 (0.1)
**Non-communicable disease**	1232 (32)
Injuries, envenomations, poisoning and toxic exposures	268 (7.0)
Respiratory diseases	221 (5.8)
Cardiovascular diseases	191 (5.0)
Neurologic disorders	169 (4.4)
Genitourinary and renal disorders	96 (2.5)
Non-communicable disease, not otherwise specified	69 (1.8)
Diseases of the gastrointestinal/digestive system	68 (1.8)
Endocrine, metabolic and immune disorders	56 (1.5)
Anemia, blood dyscrasis and coagulation disorders	41 (1.1)
Neoplasms	34 (0.9)
Mental health	12 (0.3)
Diseases of the sense organs	4 (0.1)
Skin and hair diseases	3 (0.1)
Chronic joint and spine disorders	0 (0.0)
**Other**	1556 (41)
Reproductive, maternal and newborn health	397 (10)
Integrated approach to respiratory distress and respiratory failure†	336 (8.8)
Other severe illness or injury	245 (6.4)
Integrated approach to shock†	171 (4.4)
Unspecified disease process	153 (4.0)
Post-surgical disease process	126 (3.3)
Integrated approach to altered mental status†	81 (2.1)
Nutritional deficiencies	34 (0.9)
Older adult health needs	13 (0.3)

*Overlapping categories permitted.

†“Integrated approach” is defined as management of an undifferentiated patient where the cause of their presenting signs and symptoms has not yet been determined.

### Summary of systematic reviews

The search strategy identified 60 systematic reviews. As these were not amenable to the same data extraction process as individual reports but contain important information for the field, these systematic reviews are summarised in Table S1 in the **Online Supplementary Document**.

Of the reviews identified, the most common focus areas were: critical care education and capacity building (n = 17); critical care triage, care pathways systems, clinical illness severity and/or risk stratification (n = 9); and non-invasive mechanical ventilation (n = 6). Other topics addressed included invasive mechanical ventilation, acute reperfusion therapy for ischaemic stroke and ischaemic heart disease, trauma care, management of advanced vascular access, critical care ultrasound, intravenous fluid resuscitation, massive haemorrhage control, obstetrical critical care management, and prognosis-based advanced care planning.

## DISCUSSION

This review represents a comprehensive synthesis of critical care delivery in LLMIC settings, providing an important baseline for practitioners, researchers and policymakers dedicated to improving the care of critically ill patients. This is particularly important given that the 76th World Health Assembly passed a resolution in 2023 calling for global efforts to strengthen the planning and provision of emergency, critical and operative (ECO) care services as part of universal health coverage [20].

We found that the current published literature predominantly reported critical care delivery in the in-hospital setting, however, less than half the studies reported the provision of critical care in a self-defined ICU. Specialist physicians played a major role in critical care delivery. However, general doctors, nurses, advanced nursing/midlevel practitioners and pharmacists were also frequently involved. Critical care interventions and services were found to apply to patient populations across the entire lifespan and address care needs across a broad range of disease and syndrome categories, including infectious and non-communicable diseases. Care of sepsis, respiratory infections, maternal and newborn health, injuries and cardiovascular diseases were well-represented among disease processes addressed by included studies, which align with the largest contributors to the loss of global DALYs [21]. These findings reinforce the concept that critical care occurs across a continuum within a health care system and that there is a need for integrated planning and implementation of critical care delivery across disease and population-specific programs [20].

Our results also reveal important gaps in the existing research literature. The majority of reports included in this review were observational. Only 93 randomised controlled trials that enrolled patients in LLMIC countries were identified by our search strategy, indicating a need for far more interventional research to determine which critical care interventions and services are effective in LLMIC settings [22]. Future work should analyse the key results from existing interventional studies and studies with cost-effectiveness data identified by this review to inform research, program design and policymaking [13]. Notably, over half of the included papers did not specifically state the training of care providers involved in service delivery or support teams. Given the importance of interdisciplinary care teams for delivering high-quality critical care [23], future research in this area should specifically report the care providers involved in critical care delivery and the composition of care teams in addition to the critical care intervention or service, populations served and care setting. Regarding the distribution of available literature in this field, 17 LLMICs were not reported on at all by studies meeting inclusion criteria. While data on available critical care delivery models and effective interventions from other LLMIC settings may be extrapolated to other countries, heterogeneity of economies, health care system structure, resources and disease burden likely impact the effectiveness of models for critical care delivery when implemented in different settings. For example, studies of fluid resuscitation in African adult and pediatric populations with sepsis found worse mortality rates when more aggressive fluid resuscitation protocols (akin to USA and European standards of practice) are utilised, as compared to usual care [24,25]. As such, future research should include countries and regions with a current paucity of published reports. Additionally, given that ICU care and other specialty hospital-based care is commonly clustered in urban settings, there is an imperative to ensure that future work investigates ways to describe and provide early critical care in rural, remote and austere settings [10,26-28] Finally, there were relatively few reports focusing on geriatric or maternal critical care, and future work should strive for representation of these groups to ensure their care needs are met.

This review also provides methodological advancements for research on critical care delivery. As compared to prior work defining essential emergency and critical care, the list of critical care interventions and services developed for this review takes a more inclusive approach to critical care services across a range of expected costs and technological complexity [29]. The search strategy developed for this review therefore serves to advance research in this field by creating a replicable set of search terms encompassing the breadth of potential critical care interventions and services that may be found across health care systems and across patients’ progression from pre-hospital care to inpatient care.

This review has some limitations. First, it represents the state of published, peer-reviewed literature and does not comprehensively audit all current critical care delivery models. We expect a bias toward the publication of studies taking place in ICU settings, and therefore, non-traditional critical care delivery environments, such as the pre-hospital setting, are likely under-represented in our results. It is important to note that the paucity of reports on critical care delivery in the out-of-hospital setting does not accurately represent the population-based need for such services [30,31]. This review is also limited to studies published in English, which may have affected the countries of origin of included studies. This review includes reports published prior to 2020 and therefore does not reflect changes that have occurred in response to the coronavirus disease 2019 (COVID-19) pandemic. Instead, it provides an accurate baseline representation of critical care delivery outside of the pandemic response.

## CONCLUSIONS

Critical care interventions and services are delivered widely across the health care system by individuals with many different training backgrounds and are not limited to ICU or subspecialty-trained intensivists. It is well known that countries with the fewest resources shoulder a disproportionate burden of critical illness. This review has gone a step further and detailed that the largest percentage of these patients are cared for outside of formal intensive care units. Our results support the need to ensure preparedness throughout the health care system for the recognition of and response to critical illness, in alignment with the World Health Assembly resolution on integrated emergency, critical and operative care as part of universal health coverage [20]. In addition to the actions called for by the World Health Assembly resolution, there is a desperate need for more pragmatic research regarding how to deliver the best critical care with the resources available to achieve equitable global access to quality critical care.

## Additional material


Online Supplementary Document
