# Supplementary material for: Critical care delivery across health care systems in low-income and low-middle-income country settings: A systematic review
Source: J Glob Health. 2023 Dec 1;13:04141. doi: 10.7189/jogh.13.04141 (PMC10691174; doi:10.7189/jogh.13.04141)
Supplement: Online Supplementary Document [file jogh-13-04141-s001.pdf]

Online Supplementary Document

Pubmed/MEDLINE Search Strategy

(((("Critical Care"[Mesh] OR "critical care"[tw] OR "Critical Illness"[Mesh] OR "Critical Illness"[tw] OR "Critical Care Nursing"[Mesh] OR "Critical Care Outcomes"[Mesh] OR "intensive care"[tw] OR "Intensive Care Units"[Mesh] OR "intermediate care unit"[tw] OR "step down unit"[tw] OR "Shock"[Mesh] OR "Cardiac Arrest"[tw] OR "heart arrest"[MeSH] OR "Respiratory Arrest"[tw] OR "Emergency Medicine"[Mesh] OR "Evidence-Based Emergency Medicine"[Mesh] OR "Emergency Nursing"[Mesh] OR "Emergency Treatment"[mh:noexp] OR "Advanced Trauma Life Support Care"[mesh] OR "Resuscitation"[mesh] OR "cardiopulmonary resuscitation"[tw] OR "Cardiopulmonary Resuscitation"[mesh] OR "Heart Massage"[mesh] OR "Respiration, Artificial"[mesh] OR ventilator*[ti] OR icu[ti] OR picu[ti] OR nicu[ti] OR ccu[ti] OR cpr[ti] OR "ambulances"[Mesh] OR "Emergency Service, Hospital"[mesh] OR "Poison Control Centers"[mesh] OR "Triage"[mh:noexp] OR "Hospital Rapid Response Team"[Mesh])) AND ((("Developing Countries"[Mesh] OR "Medically Underserved Area"[Mesh] OR "developing country"[tw] OR "developing countries"[tw] OR "developing nation"[tw] OR "developing nations"[tw] OR "developing population"[tw] OR "developing populations"[tw] OR "developing world"[tw] OR "less developed country"[tw] OR "less developed countries"[tw] OR "less developed nation"[tw] OR "less developed nations"[tw] OR "less developed population"[tw] OR "less developed populations"[tw] OR "less developed world"[tw] OR "lesser developed country"[tw] OR "lesser developed countries"[tw] OR "lesser developed nation"[tw] OR "lesser developed nations"[tw] OR "lesser developed population"[tw] OR "lesser developed populations"[tw] OR "lesser developed world"[tw] OR "under developed country"[tw] OR "under developed countries"[tw] OR "under developed nation"[tw] OR "under developed nations"[tw] OR "under developed population"[tw] OR "under developed populations"[tw] OR "under developed world"[tw] OR "underdeveloped country"[tw] OR "underdeveloped countries"[tw] OR "underdeveloped nation"[tw] OR "underdeveloped nations"[tw] OR "underdeveloped population"[tw] OR "underdeveloped populations"[tw] OR "underdeveloped world"[tw] OR "middle income country"[tw] OR "middle income countries"[tw] OR "middle income nation"[tw] OR "middle income nations"[tw] OR "middle income population"[tw] OR "middle income populations"[tw] OR "low income country"[tw] OR "low income countries"[tw] OR "low income nation"[tw] OR "low income nations"[tw] OR "low income population"[tw] OR "low income populations"[tw] OR "lower income country"[tw] OR "lower income countries"[tw] OR "lower income nation"[tw] OR "lower income nations"[tw] OR "lower income population"[tw] OR "lower income populations"[tw] OR "underserved country"[tw] OR "underserved countries"[tw] OR "underserved nation"[tw] OR "underserved nations"[tw] OR "underserved population"[tw] OR "underserved populations"[tw] OR "underserved world"[tw] OR "under served country"[tw] OR "under served countries"[tw] OR "under served nation"[tw] OR "under served nations"[tw] OR "under served population"[tw] OR "under served populations"[tw] OR "under served world"[tw] OR "deprived country"[tw] OR "deprived countries"[tw] OR "deprived nation"[tw] OR "deprived nations"[tw] OR "deprived population"[tw] OR "deprived populations"[tw] OR "deprived world"[tw] OR "poor country"[tw] OR "poor countries"[tw] OR "poor nation"[tw] OR "poor nations"[tw] OR "poor population"[tw] OR "poor populations"[tw] OR "poor world"[tw] OR "poorer country"[tw] OR "poorer countries"[tw] OR "poorer nation"[tw] OR "poorer nations"[tw] OR "poorer population"[tw] OR "poorer populations"[tw] OR "poorer world"[tw] OR "developing economy"[tw] OR "developing economies"[tw] OR "less developed economy"[tw] OR "less developed economies"[tw] OR "lesser developed economy"[tw] OR "lesser developed economies"[tw] OR "under developed economy"[tw] OR "under developed economies"[tw] OR "underdeveloped economy"[tw] OR "underdeveloped economies"[tw] OR "middle income economy"[tw] OR "middle income economies"[tw] OR "low income economy"[tw] OR "low income economies"[tw] OR "lower income economy"[tw] OR "lower income economies"[tw] OR "low gdp"[tw] OR "low gnp"[tw] OR "low gross domestic"[tw] OR "low gross national"[tw] OR "lower gdp"[tw] OR "lower gnp"[tw] OR "lower gross domestic"[tw] OR "lower gross national"[tw] OR lmic[tw] OR lmics[tw] OR "third world"[tw] OR "lami country"[tw] OR "lami countries"[tw] OR "transitional country"[tw] OR "transitional countries"[tw])) OR (Afghanistan[tw] OR "guatemala" [tw] OR "samoa" [tw] OR tonga OR "cape verde" [tw] OR Armenia [tw] OR Bangladesh[tw] OR Benin[tw] OR Bhutan[tw] OR Bolivia[tw] OR "Burkina Faso"[tw] OR Burundi[tw] OR "Cabo Verde"[tw] OR Cambodia[tw] OR Cameroon[tw] OR "Central African Republic"[tw] OR Chad[tw] OR Comoros[tw] OR Congo[tw] OR "Cote d'Ivoire"[tw] OR Djibouti[tw] OR Egypt[tw] OR "El Salvador"[tw] OR Eritrea[tw] OR Ethiopia[tw] OR Gambia[tw] OR Ghana[tw] OR Guinea[tw] OR "Guinea-Bissau"[tw] OR Haiti[tw] OR Honduras[tw] OR India[tw] OR Indonesia[tw] OR Kenya[tw] OR Kiribati[tw] OR "Democratic People's Republic of Korea"[tw] OR "north korea"[tw] OR Kosovo[tw] OR

Kyrgyzstan[tw] OR laos[tw] OR Lesotho[tw] OR Liberia[tw] OR Madagascar[tw] OR Malawi[tw] OR Mali[tw] OR Mauritania[tw] OR Micronesia[tw] OR Moldova[tw] OR Mongolia[tw] OR Morocco[tw] OR Mozambique[tw] OR Myanmar[tw] OR Nepal[tw] OR Nicaragua[tw] OR Niger[tw] OR Nigeria[tw] OR Pakistan[tw] OR "Papua New Guinea"[tw] OR Philippines[tw] OR Rwanda[tw] OR "Sao Tome"[tw] OR Principe[tw] OR Senegal[tw] OR "Sierra Leone"[tw] OR "Solomon Islands"[tw] OR Somalia[tw] OR "South Sudan"[tw] OR "Sri Lanka"[tw] OR Sudan[tw] OR Swaziland[tw] OR syria[tw] OR Tajikistan[tw] OR Tanzania[tw] OR "Timor-Leste"[tw] OR Togo[tw] OR Tunisia[tw] OR Uganda[tw] OR Ukraine[tw] OR Uzbekistan[tw] OR Vanuatu[tw] OR Vietnam[tw] OR "West Bank"[tw] OR Yemen[tw] OR Zambia[tw] OR Zimbabwe[tw] OR Kyrgyz[tw] OR Syrian[tw] OR "lao pdr"[tw] OR Burma[tw] OR gaza[tw] OR "ivory coast"[tw] OR Afghanistan[mesh] OR "guatemala" [mesh] OR "samoa" [mesh] OR tonga OR "cape verde" [mesh] OR Armenia [mesh] OR Bangladesh[mesh] OR Benin[mesh] OR Bhutan[mesh] OR Bolivia[mesh] OR "Burkina Faso"[mesh] OR Burundi[mesh] OR "Cabo Verde"[mesh] OR Cambodia[mesh] OR Cameroon[mesh] OR "Central African Republic"[mesh] OR Chad[mesh] OR Comoros[mesh] OR Congo[mesh] OR "Cote d'Ivoire"[mesh] OR Djibouti[mesh] OR Egypt[mesh] OR "El Salvador"[mesh] OR Eritrea[mesh] OR Ethiopia[mesh] OR Gambia[mesh] OR Ghana[mesh] OR Guinea[mesh] OR "Guinea-Bissau"[mesh] OR Haiti[mesh] OR Honduras[mesh] OR India[mesh] OR Indonesia[mesh] OR Kenya[mesh] OR Kiribati[mesh] OR "Democratic People's Republic of Korea"[mesh] OR Kosovo[mesh] OR Kyrgyzstan[mesh] OR laos[mesh] OR Lesotho[mesh] OR Liberia[mesh] OR Madagascar[mesh] OR Malawi[mesh] OR Mali[mesh] OR Mauritania[mesh] OR Micronesia[mesh] OR Moldova[mesh] OR Mongolia[mesh] OR Morocco[mesh] OR Mozambique[mesh] OR Myanmar[mesh] OR Nepal[mesh] OR Nicaragua[mesh] OR Niger[mesh] OR Nigeria[mesh] OR Pakistan[mesh] OR "Papua New Guinea"[mesh] OR Philippines[mesh] OR Rwanda[mesh] OR "Sao Tome and Principe"[mesh] OR Senegal[mesh] OR "Sierra Leone"[mesh] OR "melanesia"[mesh] OR Somalia[mesh] OR "South Sudan"[mesh] OR "Sri Lanka"[mesh] OR Sudan[mesh] OR Swaziland[mesh] OR syria[mesh] OR Tajikistan[mesh] OR Tanzania[mesh] OR "Timor- Leste"[mesh] OR Togo[mesh] OR Tunisia[mesh] OR Uganda[mesh] OR Ukraine[mesh] OR Uzbekistan[mesh] OR Vanuatu[mesh] OR Vietnam[mesh] OR Yemen[mesh] OR Zambia[mesh] OR Zimbabwe[mesh]))) NOT ("Emergency Services, Psychiatric"[Mesh] OR "Addresses"[pt] OR "Autobiography"[pt] OR "Bibliography"[pt] OR "Biography"[pt] OR "Case Reports"[pt] OR "Comment"[pt] OR "Congresses"[pt] OR "Consensus Development Conference"[pt] OR "Consensus Development Conference, NIH"[pt] OR "Dictionary"[pt] OR "Directory"[pt] OR "Editorial"[pt] OR "Electronic Supplementary Materials"[pt] OR "Festschrift"[pt] OR "Historical Article"[pt] OR "Interactive Tutorial"[pt] OR "Interview"[pt] OR "Lectures"[pt] OR "Legal Cases"[pt] OR "Legislation"[pt] OR "Letter"[pt] OR "News"[pt] OR "Newspaper Article"[pt] OR "Overall"[pt] OR "Patient Education Handout"[pt] OR "Periodical Index"[pt] OR "Personal Narratives"[pt] OR "Portraits"[pt] OR "Practice Guideline"[pt] OR "Published Erratum"[pt] OR "Retracted Publication"[pt] OR "Retraction of Publication"[pt] OR "Technical Report"[pt] OR "Video-Audio Media"[pt] OR "Webcasts"[pt] OR "case report"[ti] OR bystand*[ti])) AND english[lang] NOT ("animals"[mesh] NOT "humans"[mesh]) AND ("2008/01/01"[PDAT] : "3000/12/31"[PDAT])

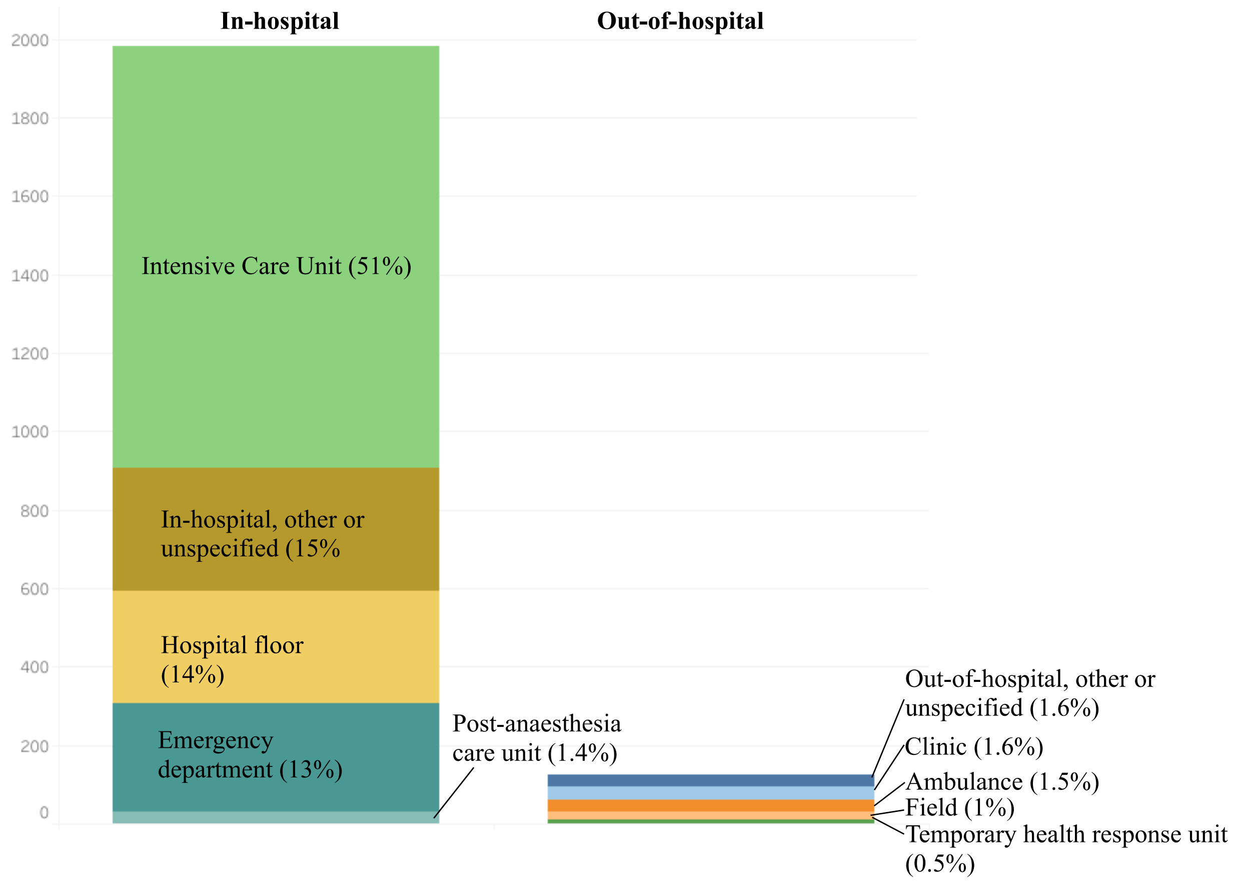

Figure S1: Care settings reported by included studies
Multiple categories permitted per reference.

Table S1: Summary of systematic reviews

First Author	Year of Publication	Journal	PubMed ID	Title	Search period	Countries involved in review	Summary
**Acute reperfusion therapy: medical or interventional (neurologic, ischemic stroke, coiling)**							
Pandian	2017	Neuroepidemiology	28848165	Strategies to Improve Stroke Care Services on Low- and Middle-Income Countries: A Systematic Review	1966-2015	Multiple LMICs included	The authors found several reports on evidence-based implementable stroke services in LMICs. Some strategies are economic, feasible and reproducible but remain untested. Data on effects on outcomes and sustainability of changes were very limited. Further research of implementation of locally and regionally adapted stroke services should be prioritized.
**Advanced trauma resuscitation/ATLS/trauma care checklist use**							

Choi	2017	Emerg Med Australas	28782875	Comparison of trauma Care systems in Asian countries: a systematic literature review	2005-2014	Pakistan, India, Vietnam, Indonesia, Thailand, Iran, Malaysia, China	Trauma care systems were poorly developed and unorganised in most of the selected 13 Asian countries, with the exception of a few highly developed countries.
Reynolds	2017	Annu Rev Public Health	28125389	The Impact of Trauma Care Systems in Low- and Middle-Income Countries	Inception-2016	Multiple LMICs included	This review has identified and described reports that evaluated the impact of trauma care systems and system components in LMICs. Although we identified 71 reports, the majority of LMICs had no reports at all. Reports that described the results of trauma care training, prehospital system establishment, overall system organization, and improvements to the availability of specialty care were more commonly reported than other system components. These findings suggest several priority areas for research, program development, and funding. Specifically, there are a number of low-cost, high-value-added organizational interventions that involve only minimal input of new material resources. Quality-improvement, costing, rehabilitation, and legislation and governance are particularly neglected areas.
Henry	2012	J Trauma Acute Care Surg	22743393	Prehospital trauma systems reduce mortality in developing countries: A systematic review and meta-analysis	Inception-2010	Multiple LMICs included	Prehospital trauma systems in developing countries, particularly middle-income countries, reduce mortality. These data should inform and encourage developing countries to adopt prehospital trauma systems at the policy level.

Forrester	2019	Disaster Med Public Health Prep	3120383	The Golden Hour After Injury Among Civilians Caught in Conflict Zones	1990-2017	Yugoslavia/Bosnia/Herzegovina, Syria, Afghanistan, Iraq, Israel, Cambodia, Somalia, Georgia, Lebanon, Nigeria, Democratic Republic of Congo, and Turkey	Anticipated transport times have important implications for field triage of injured persons in civilian conflict settings because existing overburdened civilian health care systems may become further overwhelmed if in-hospital health capacity is unable to keep pace with inflow of the severely wounded.
**Advanced vascular access**							
Ista	2016	Lancet Infectious Disease	26907734	Effectiveness of insertion and maintenance bundles to prevent central-line-associated bloodstream infections in critically ill patients of all ages: a systematic review and meta-analysis	1990-2015	Multiple LMICs included	Implementation of central-line bundles has the potential to reduce the incidence of CLABSIs.
**Critical Care Education/Capacity-Building**							
Opiyo	2010	Cochrane Database Syst Rev	25968066	In-service training in the care of the seriously ill newborn or child for health professionals in developing countries	1950-2009	Kenya, Sri Lanka	Although there is some evidence of benefit, there is limited evidence that in-service neonatal emergency care courses improve health-care workers' practices when caring for a seriously ill newborn.
Nowacki	2013	Int J Emerg Med	23866095	A review of published literature on emergency medicine training programs in low- and middle-income countries	1948-2011	Malaysia, Turkey, South Africa, Peru, Armenia, Cameroon, China, Costa Rica, Ecuador, Jordan, Papua New Guinea	The review found that EM training is still in its infancy in resource-limited health systems. Only a few detailed reports exist on these programs' successes and failures, including efforts to improve graduate retention. Despite the lack of published data on the development of EM residency training programs in these settings, the authors make specific recommendations to help guide future authors on reporting on such efforts.

Mendhi	2018	Women Birth	29793845	Review of educational interventions to increase traditional birth attendants' neonatal resuscitation self-efficacy	1980 to 2017	Argentina, Congo, Guatemala, India, Indonesia, Pakistan, Kenya, Zambia, and Ethiopia	Culturally appropriate methods, such as role-play, demonstration, and pictorial charts, can contribute to increased knowledge and self-efficacy related to neonatal resuscitation. A team approach to training traditional birth attendants, assisted by village health workers during home-based childbirths may reduce neonatal mortality rates.
Murthy	2015	Plos One	25617837	Intensive Care Unit capacity in low-income countries: a systematic review	2004-2014	15 LMICs	Low-income countries lack ICU beds, and more than 50% of these countries lack any published data on ICU capacity. Most ICUs in low-income countries are located in large referral hospitals in cities.
Pammi	2016	Neonatology	27222260	Newborn resuscitation training programmes reduce Early neonatal mortality	Inception to 2015	Multiple LMICs	Standardised formal neonatal resuscitation training in low- and middle-income countries reduces early neonatal mortality, but its effects on birth asphyxia and neurodevelopmental outcomes remain uncertain.
Obermeyer	2015	Bull World Health Organ	26478615	Emergency care in 59 low- and middle-income countries: a systematic review	1990-2014	59 LMICs	Available data on emergency care in LMICs indicate high patient loads and mortality, particularly in sub-Saharan Africa, where a substantial proportion of all deaths may occur in emergency departments. The combination of high volume and the urgency of treatment make emergency care an important area of focus for interventions aimed at reducing mortality in these settings.

Meaney	2010	Resuscitation	20727656	Resuscitation training in developing countries: a systematic review	1990-2008	Multiple LMICs	Resuscitation training in developing countries was well received and viewed as valuable training by the students and local counterparts. Important student and training environment characteristics as well as important outcomes (cognitive, psychomotor skill competence, operational performance, patient outcome and costeffectiveness) were inconsistently defined and reported. Courses often were modified to easily available training resources rather than the needs and resources of the local healthcare environment.
Callese	2015	J Surg Res	25450600	Trauma system Development in low- and middle-income countries: a review	NA	Multiple LMICs	There is no single model of emergency medical services and trauma services (EMS and TS) in low- and middle-income countries (LMICs). Successful EMS and TS systems share common characteristics. A predevelopment needs assessment is critical in identifying existing EMS and TS resources as a foundation for further development. Implementation requires coordination of preexisting resources with cost-effective initiatives that involve local stakeholders.
Dempsey	2015	Cochrane Database Syst Rev	26337958	Standardised formal resuscitation training programmes for Reducing mortality and morbidity in newborn infants	Inception-2015	Multiple LMICs included	Standardised formal resuscitation training (SFNRT) compared to basic newborn care or basic newborn resuscitation, in developing countries, results in a reduction of early neonatal and 28-day mortality. Randomised trials of SFNRT should report on neonatal morbidity including hypoxic ischaemic encephalopathy and neurodevelopmental outcomes. Innovative educational methods that enhance knowledge and skills and teamwork behaviour should be evaluated.

Baker	2017	Journal of Critical Care	29174463	Critical care of tropical disease in low income countries: Report from the Task Force on tropical Diseases by the World Federation of Societies of Intensive and critical Care medicine	NA	Multiple LMICs included	Tropical disease results in a great burden of critical illness. The same life-saving and supportive therapies to maintain vital organ functions that comprise critical care are required by these patients as for all other diseases. In low income countries, the little available data points towards high mortality rates and big challenges in the provision of critical care. Improving critical care in low income countries requires a focus on hospital design, training, triage, monitoring & treatment modifications, the basic principles of critical care, hygiene and the involvement of multi-disciplinary teams. As a large proportion of critical illness from tropical disease is in low income countries, the impact and reductions in mortality rates of improved critical care in such settings could be substantial.
Baker	2009	Trop Med Int Health	19207174	Critical Care in low-income countries	NA	Multiple LMICs included	Improved critical care could have a significant effect on the burden of disease and effects of ill health. Research into the most cost-effective treatments and methods of caring for critically ill patients is urgently needed.
Anderson	2018	Resuscitation	29545135	The train the trainer model for the propagation of resuscitation Knowledge in limited resource settings: a systematic review	Inception-2018	Multiple LMICs included	While the TTT model of programming for the dissemination of resuscitation education is promising, further research is necessary especially relating to sustainability and impact on patient outcomes. Familiarity with the local environment, language, culture, resources and economic realities prior to the initiation of programming is key to success.

Zaka	2018	Implementation science	29370845	Quality improvement initiatives for hospitalised small and sick newborns in low- and middle-income countries: a systematic review	2000-2017	Multiple LMICs included	The frequency of meso level, single centre, and educational interventions suggests that these interventions may be easier for programme planners to implement. The success of some interventions in reducing morbidity and mortality rates suggests that QI approaches have a high potential for benefit to newborns.
Reisman	2016	Pediatrics	27388500	Newborn Resuscitation Training in Resource-Limited Settings: A Systematic Literature Review	NA	Multiple LMICs included	Knowledge and skills falloff is a significant barrier to the success of NR training programs and possibly to reducing newborn mortality in LMICs. Refresher training and structured practice show significant promise. Additional research is needed to implement and assess retention improvement strategies in classroom and clinical settings.
Lee	2011	BMC Public Health	21501429	Neonatal resuscitation and immediate newborn assessment and stimulation for the prevention of neonatal deaths: a systematic review, meta-analysis and Delphi estimation of mortality effect	1980-2010	Multiple LMICs included	Neonatal resuscitation training in facilities reduces term intrapartum-related deaths by 30%. Yet, coverage of this intervention remains low in countries where most neonatal deaths occur and is a missed opportunity to save lives. Expert opinion supports smaller effects of neonatal resuscitation on preterm mortality in facilities and of basic resuscitation and newborn assessment and stimulation at community level. Further evaluation is required for impact, cost and implementation strategies in various contexts.

Mould-Millman	2019	Afr J Emerg Med	30873352	Fifteen years of emergency medicine literature in Africa: A scoping review	1999-2014	African LMICs	Review revealed a considerable increase in the growth of African EM literature from 1999 to 2014. Overwhelmingly, articles were observational, studied all-comers, and focused on undifferentiated complaints. The articles discovered in this scoping review are reflective of the relatively immature and growing state of African EM.
Ljungblad	2019	International Journal of Africa Nursing Sciences	NA	The impact of skilled birth attendants trained on newborn resuscitation in Tanzania: A literature review	Inception-2016	Tanzania	No training is stronger than its weakest factor, and extensive challenges in Tanzania are reported at several levels. The reported challenges are lack of human resources, knowledge, supplies, humanization and dignity in maternity wards. Additionally, hospitals often have neither water nor soap. Further training, action and research are needed to improve skilled birth attendant's possibilities to save millions of newborn lives.
**Critical care triage / care pathways systems / clinical illness severity and/or risk stratification**							
Ryan	2016	J Obstet Gynaecol Can	27720089	The Usefulness of the APACHE II score in obstetric critical Care: a structured review	1985-2015	Multiple LMICs	The APACHE II score consistently overestimates mortality risks for pregnant and recently pregnant women receiving critical care, whether they reside in HICs or LMICs. There is a need for a pregnancy-specific outcome prediction model for these women.
Pollock	2010	Intensive Care Med	20631987	Pregnant and postpartum admissions to the Intensive Care Unit: a systematic review	1990-2008	Multiple LMICs	ICU admission of pregnant and postpartum women occurs infrequently, with obstetric conditions responsible for the majority of ICU admissions. The ICU admission profile of women was similar in developed and developing countries; however, the maternal mortality rate remains higher for ICUs in developing countries, supporting the need for ongoing service delivery improvements.

Hansoti	2017	BMC Pediatrics	28122537	Reliability and Validity of Pediatric Triage tools evaluated in low resource settings: a systematic review	2000-2015	Multiple LMICs	Studies evaluating triage tools for pediatric patients in low and middle income countries are scarce. Furthermore the methodology utilized in the conduct of these studies varies greatly and does not allow for the comparison of tools across study sites.
Haniffa	2018	Critical care	29373996	Performance of critical Care prognostic Scoring systems in low and middle-income countries: a systematic review	Inception-2007	India, Thailand, Brazil, Iran, Bangladesh, Indonesia, Pakistan, Philippines	Robust interpretations regarding the applicability of prognostic models are currently hampered by poor adherence to reporting guidelines, especially when reporting missing value handling. Performance of mortality risk prediction models in LMIC ICUs is at best moderate, especially with limitations in calibration. This necessitates continued efforts to develop and validate LMIC models with readily available prognostic variables, perhaps aided by medical registries.
Jenson	2018	Eur J Emerg Med	28263204	Reliability and Validity of emergency department Triage tools in low- and middle-income countries: a systematic review	2000-2015	Multiple LMICs	The quality of evidence supporting any single triage tool's validity and reliability in LMICs is moderate at best. Research on triage tool applicability in low-resource environments must be targeted to the actual clinical environment where the tool will be utilized, and must include low-income countries and rural, primary care settings.

Aluvaala	2017	Systematic reviews	29212522	A systematic review of neonatal treatment intensity scores and their potential application in low-resource setting Hospitals for predicting mortality, morbidity and estimating resource use	Inception-2016	Multiple LMICs included	Existing neonatal treatment intensity models show promise in predicting mortality and morbidity. There is however low certainty in the evidence on their performance in essential neonatal care in low resource settings as all studies had methodological limitations and were conducted in intensive care. The approach may however be developed further for low resource settings like Kenya because treatment data may be easier to obtain compared to measures of physiological status.
Tan	2019	JAMA Pediatrics	30742207	Global Case-Fatality Rates in Pediatric Severe Sepsis and Septic Shock: A Systematic Review and Meta-analysis	Inception-2017	Multiple LMICs included	Despite the declining trend of pediatric severe sepsis and septic shock CFRs, the disparity between developing and developed countries persists. Further characterizations of vulnerable populations and collaborations between developed and developing countries are warranted to reduce the burden of pediatric sepsis globally.
Murthy	2019	Plos One	31022223	Risk factors of neonatal sepsis in India: A systematic review and meta-analysis	Inception-2018	India	Male neonates, outborn admissions, need for artificial ventilation, gestational age <37 weeks and premature rupture of membranes are risk factors for sepsis among neonates in India. Robustly designed and reported research is urgently needed to confirm the role of other risk factors of neonatal sepsis in India.

Manoochehry	2019	Arch Acad Emerg Med	30847441	A Comparison between the Ability of Revised Trauma Score and Kampala Trauma Score in Predicting Mortality; a Meta-Analysis	Inception-2018	Iran, Uganda, Nigeria, Colombia, Turkey	However, regarding accuracy and performance, RTS was better than KTS for distinguishing between mortality and survival; both of them are beneficial trauma scoring tools which can be used in LMICs. Further studies are required to specify the appropriate choice of the RTS or KTS regarding the type of injury and different conditions of the patient.
**Critical care ultrasound**							
Becker	2016	Trop Med Int Health	26683523	The use of portable ultrasound devices in low- and middle-income countries: a systematic review of the literature	Inception-2015	Multiple LMICs included	This review highlights the potential for portable ultrasound devices in LMIC, and hand-held machines in particular. However, so far the lack of sufficient data pertaining to the role of portable ultrasound systems for improved management of certain clinical syndromes does not allow for general practice recommendations. Authors conclude that there is a need to scientifically assess and evaluate the use of these devices in LMIC at a large scale to better define the scope, impact and potential benefits of hand-carried ultrasound in resource-constrained settings worldwide.
Hallweaver	2019	Curr Radiol Rep	NA	Ultrasound in the limited-resource setting: A systematic qualitative review	Inception-2018	Multiple LMICs included	US continues to be a highly utilized tool in limited-resource settings. We identified several common themes among the recent literature: the use of educational interventions to train non-physician providers, the use of US for improved screening methods, and the expanded role of US in clinical purposes including Infectious Disease, Echocardiography, Pulmonary, and Obstetrics.
**Intravenous fluid resuscitation**							

Opiyo	2014	BMJ Open	24785400	Immediate fluid Management of children with severe febrile illness and signs of impaired circulation in low-income settings: a contextualised systematic review	Inception to 2014	Brazil, India, Kenya, Tanzania and Uganda	This study shows that in LMICs fluid boluses increase mortality in children with severe febrile illness and impaired circulation, and this increased risk is consistent across children with severe and less severe circulatory impairment.
Li	2018	Cochrane Database Syst Rev	30536956	Liberal versus conservative fluid therapy in adults and children with sepsis or septic shock	Inception until 2018	India, Uganda, Kenya, Tanzania	Low- to high-quality evidence indicates that liberal fluid therapy might increase mortality among children with sepsis or septic shock in hospital and at four-week follow-up.
**Massive hemorrhage control**							
Ker	2012	BMC Emergency Medicine	22380715	Avoidable mortality from giving tranexamic acid to bleeding trauma patients: an estimation based on WHO mortality data, a systematic literature review and data from the CRASH-2 trial	2004-2011	Multiple LMICs	The use of TXA in the treatment of traumatic bleeding has the potential to prevent many premature deaths every year. A large proportion of the potential health gains are in low and middle income countries.
**Mechanical ventilation, invasive**							
Fathy	2013	Egyptian Journal of Chest Diseases and Tuberculosis	NA	Analysis of ventilator associated pneumonia (VAP) studies in Egyptian University Hospitals	2003-2013	Egypt	Risk factors for VAP included leukopenia, thrombocytopenia, high CRP, metabolic acidosis, nasal endotracheal intubation, re-intubation, prior antibiotic use, contaminated ICU environment with inadequate infection control, antacid and H2 blocker use, coma, and coma-like symptoms. Acinetobacter, Candida, and Pseudomonas aerogenosa were the most common causative organisms.
Bonell	2018	Clin Infect Dis	29982303	A systematic review and meta-analysis of ventilator associated pneumonia in adults in Asia; an analysis of national income level on incidence and etiology.	1990-2017	Multiple LMICs	VAP remains a common cause of HAI, especially in low- and middle-income countries, and antibiotic resistance is high.

Arabi	2008	Int J Infect Dis	18502674	Ventilator-Associated Pneumonia in adults in developing countries: a systematic review	1966-2007	Multiple LMICs included	Based on the existing literature, the rate of VAP in developing countries is higher than NHSN benchmark rates and is associated with a significant impact on patient outcome. Only a few studies reported successful interventions to reduce VAP. There is a clear need for additional epidemiologic studies to better understand the scope of the problem. Additionally, more work needs to be done on strategies to prevent VAP, probably with emphasis on practical, low-cost, low technology, easily implemented measures.
Aelami	2014	Antimicrobial Resistance & Infection Control	NA	Ventilator-associated pneumonia in neonates, infants and children	Inception-2014	Multiple LMICs included	Hand hygiene, glove and gown use for endotracheal tube manipulation, backrest elevation, oral care with chlorhexidine, stress ulcer prophylaxis, cuff pressure maintenance where appropriate, use of orogastric tubes, avoidance of gastric overdistension, and elimination of nonessential tracheal suction pevented VAP. When applied as a multimodal strategy by an interdisciplinary team, these interventions are most likely to be successful among neonates, infants, and children, and have proven effectiveness in high-, as well as in low- and middle-income countries.
**Mechanical ventilation, non-invasive**							

Ekhaguere	2018	Paediatr Respir Rev	29907334	Risk and benefits of Bubble Continuous Positive Airway Pressure for neonatal and childhood respiratory diseases in Low- and Middle-Income countries	NA	Malawi, Bangladesh, Ghana	Bubble CPAP is a safe and cheap therapy. Although the LMIC evidence is weak, combined with HIC evidence, bCPAP reduces mortality, eliminates mechanical ventilation, and prevents extubation failure. Its low cost and low technological requirements have made it popular in low-income countries. However, compared to high-income countries' standards of care, its use may be suboptimal. Standard low-cost devices and high-quality research studies on bCPAP implementation in LMICs are required.
Mandelzweig	2018	J Crit Care	29426584	Non-invasive ventilation in children and adults in low- and low-middle income countries: A systematic review and meta-analysis	Inception until 2016	India, Pakistan, Nepal, Egypt, Palestine, Morocco, Vietnam, Ghana, Malawi, Bangladesh	Non invasive ventilation appeared to be safe in pediatric and adult patients with acute respiratory failure and in the peri-extubation period in these resource-constrained settings
Martin	2014	Arch Dis Child Fetal Neonatal Ed	25085942	Efficacy and safety of Bubble CPAP in neonatal Care in low and middle income countries: a systematic review	1946-2014	Iran, India	There is evidence that bubble CPAP is safe and reduces the need for mechanical ventilation. Further research into the efficacy of bubble CPAP in low-income and middle-income countries is needed.
Gupta	2015	Indian Pediatr	25929631	Continuous positive Airway pressure in preterm neonates: an update of current Evidence and implications for developing countries	Inception-2014	Multiple LMICs	CPAP, if used early and judiciously, is an effective intervention and need immediate scaling-up in resource-limited settings. Future research should focus on the ideal interface and the CPAP delivery methods.
Won	2019	Paediatr Int Child Health	30375281	Bubble CPAP devices for infants and children in resource-limited settings: review of the literature	Inception-2017	Multiple LMICs included	This narrative review found many studies which evaluated the real clinical outcomes with bCPAP in the target population as well as technical comparison of bCPAP components. However, many studies were not blinded or randomised and there was significant heterogeneity in design and outcome measures.

Jat	2019	Cochrane Database Syst Rev	30701528	Continuous positive airway pressure (CPAP) for acute bronchiolitis in children	1970-2017	India	Limited, low-quality evidence suggests that breathing improved in children with bronchiolitis who received CPAP. Larger, adequately powered trials are needed to evaluate the effect of CPAP for children with acute bronchiolitis.
	**Multiple interventions/services**						
Becker	2017	Acad Emerg Med	28474823	Global Emergency Medicine: A Review of the Literature From 2016	2016	Multiple LMICs included	In 2016, studies and reviews with a focus on infectious diseases, pediatrics, and the use of ultrasound in resource-limited settings represented the majority of articles selected for final review.
Becker	2016	Acad Emerg Med	27146277	Global Emergency Medicine: A Review of the Literature From 2015	2015	Multiple LMICs included	In 2015, there were almost twice as many articles found compared to the 2014 review. As in prior years, the majority of articles focused on infectious diseases.
Morton	2018	Int J Environ Res Public Health	30223556	The Early Recognition and Management of Sepsis in Sub-Saharan African Adults: A Systematic Review and Meta-Analysis	2000-2018	Sub-Saharan LMICs	There is poor availability of literature on protocolized management of sepsis in subsaharan africa
Dahn	2018	Int J Crit Illn Inj Sci	30181970	Acute Care for the three leading causes of mortality in lower-middle-income countries: a systematic review	Inception-2017	Multiple LMICs included	Acute care of patients with IHD, stroke, and LRI in low-and middle-income countries has been studied. There are many studies demonstrating feasibility and benefit of acute care interventions in these settings including but not limited to percutaneous coronary intervention, stroke-specific ward care, and improved mortality and decreased morbidity in particular patients with oxygen therapy or NIV with LRI.
**Obstetric Critical Care Management**							

Bunch	2018	BMJ Open	30573478	Systematic review to investigate the safety of induction and augmentation of labour among pregnant women with iron-deficiency anaemia	Inception-2018	India	The best approach is to prevent anaemia, but a large number of women in low-to-middle-income countries present with severe anaemia during labour. In such women, appropriate peripartum management could prevent complications and death. Our review showed that at present we do not know if induction and augmentation of labour is safe in pregnant women with iron-deficiency anaemia and further research is required.
Alanazy	2019	Int J Environ Res Public Health	31100851	Factors Impacting Patient Outcomes Associated with Use of Emergency Medical Services Operating in Urban Versus Rural Areas: A Systematic Review	1988 to 2018	India	The Janani Express Yojana (JEY) was India's first emergency transportation model dedicated exclusively to transporting mothers giving birth. This innovative service has been well utilized particularly by mothers in rural areas.
**Oxygen delivery, high-flow**							
Lin	2019	Arch Dis Child	30655267	High-flow nasal cannula therapy for children with bronchiolitis: a systematic review and meta-analysis	Inception-2018	India, China	The systematic review suggests HFNC is safe as an initial respiratory management, but the evidence is still lacking to show benefits for children with bronchiolitis compared with SOT or nCPAP.
**Prognosis-based advance care planning**							
Kim	2019	J Palliat Med	30632880	End-of-Life Care in Neonatal Intensive Care Units from an Asian Perspective: An Integrative Review of the Research Literature	2007-2016	Hong Kong, India, Israel, Japan, Mongolia, Taiwan, and Turkey	While infant EOL care practice and research vary by country, few articles address these matters in Asia. This integrative review characterizes infant EOL care in Asia and explores cultural influences on such care.
**Support of hemodynamic instability and management of acute life-threatening organ dysfunction**							

Menon	2013	Pediatr Crit Care Med	23867428	A systematic review and meta-analysis on the Effect of steroids in Pediatric shock	1994-2012	Multiple LMICs	The literature on the use of steroids in pediatric shock is limited in amount and methodological quality and demonstrates conflicting results. This systematic review documents the very limited evidence on which current guidelines in this area are based and strongly supports the need for a well-designed, pragmatic randomized controlled trial on the use of steroids in pediatric shock to inform future guidelines.
**Support of respiratory insufficiency/failure**							
Woldeamanuel	2016	J Neurol Sci	27538652	Case fatality of adult tetanus in Africa: systematic review and meta-Analysis	Inception until 2016	Nigeria, Ethiopia, Ghana, Uganda, Senegal, Democratic Republic of Congo, and Tanzania	Despite declining tetanus incidence rates, case-fatality is still high in African care facilities. High rates of tetanus case fatality indicate lower quality of medical care at hospital settings.Most common causes of death are complication arising from dysautonomia and respiratory arrest secondary to laryngospasm.
Sankar	2016	J Perinatol	27109091	Efficacy and safety of surfactant replacement therapy for preterm neonates with respiratory distress syndrome in low- and middle-income countries: a systematic review	1998-2013	Multiple LMICs	Surfactant therapy is effective, safe and feasible in level-3 neonatal units and has the potential to reduce neonatal mortality and air leaks in low-resource settings
**Treatment of severe infections/inflammation other than IV fluids and antibiotics**							
Soares-Weiser	2010	BMC Infect Dis.	20626907	Ribavirin for Crimean-Congo hemorrhagic fever: systematic review and meta-Analysis	Inception until 2009	Pakistan, Iran, Turkey, Russia	Ribavirin is not shown to improve outcome in Crimean-Congo hemorrhagic fever patients
